# Lab-to-Field Transition in FBG Sensors: A Quantitative Assessment of Literature-Reported Deployment Maturity

**DOI:** 10.3390/s26144403

**Published:** 2026-07-10

**Authors:** Alaa N. D. Alhussein, Mohammad R. Radi, Sergey V. Kuryntsev, Bulat I. Valeev, Timur A. Agliullin, Oleg G. Morozov, Airat Zh. Sakhabutdinov

**Affiliations:** 1 Department of Radiophotonics and Microwave Technologies, Kazan National Research Technical University Named After A. N. Tupolev-KAI, 10, K. Marx St., Kazan 420111, Russia; bivaleev@kai.ru (B.I.V.); taagliullin@kai.ru (T.A.A.); ogmorozov@kai.ru (O.G.M.); azhsakhabutdinov@kai.ru (A.Z.S.); 2 Information Technology Research and Development Centre, University of Kufa, P.O. Box 21, Kufa 540011, Najaf, Iraq; mohammad@uokufa.edu.iq; 3 Department of Materials Science and Welding, Kazan National Research Technical University Named After A. N. Tupolev-KAI, 10, K. Marx St., Kazan 420111, Russia; svkuryntsev@kai.ru

**Keywords:** fiber Bragg grating (FBG), structural health monitoring (SHM), lab-to-field transition, optical fiber sensors, harsh environment sensing, biomedical sensing, industrial monitoring, chemical sensing, deployment challenges, multi-parameter sensing

## Abstract

Fiber Bragg Grating (FBG) sensors have become an important sensing technology owing to their high sensitivity, multiplexing capability, and ability to operate in demanding environments. Despite extensive research activity, the transition of FBG sensing systems from laboratory studies to practical deployment remains uneven across application domains. This review examines that transition through a multi-level assessment combining publication trends, deployment maturity, application domains, and sensing performance. The analysis of publications from 2021 to 2026 shows that most of the reported systems remain at the laboratory-validation stage, while examples of short-term field deployment and long-term operational deployment remain comparatively limited. Marked differences were observed between various application domains. Structural health monitoring and several harsh-environment applications provide the strongest evidence of literature-reported field deployment, whereas biomedical and chemical sensing systems remain concentrated at lower maturity levels despite intensive research activity. The results suggest that deployment maturity is influenced by the relationship between the sensing mechanism and the target measurand, integration requirements, environmental robustness, system complexity, and application demand. Overall, the findings indicate that the principal challenge facing FBG sensing technology is no longer sensing performance itself, but the ability to achieve reliable, scalable, and sustainable operation under real service conditions.

## 1. Introduction

Fiber Bragg Grating sensors are among the most widely studied optical sensing technologies because of their high sensitivity, multiplexing capability, compact size, and immunity to electromagnetic interference [1]. Over the past decade, advances in sensor design, interrogation techniques, and signal processing have expanded their use across structural health monitoring, biomedical systems, industrial monitoring, chemical sensing, and harsh-environment applications [2]. These developments have led to substantial improvements in measurement accuracy, sensitivity, and multi-parameter sensing capabilities.

FBG sensors have demonstrated significant potential for monitoring strain, temperature, pressure, and vibration, even in complex systems such as bridges, pipelines, aerospace structures, and medical devices [3,4,5]. Industrial and environmental applications have also demonstrated the adaptability of FBG sensing systems to demanding operating conditions, including chemically aggressive environments as well as elevated temperatures and pressures [6,7,8]. Taken together, these achievements may suggest that FBG sensing technology has reached a high level of technical maturity.

Despite substantial advances in FBG sensing technologies, their practical deployment remains uneven across application domains. Many reported systems are validated under controlled laboratory conditions, whereas pilot-scale studies, short-term field deployments, and long-term operational demonstrations remain comparatively limited [8,9]. This contrast indicates that high sensing performance alone does not necessarily imply deployment maturity.

A systematic assessment is therefore needed to determine how far FBG sensing systems have progressed from laboratory validation toward practical use. Such an assessment should link research activity with deployment maturity, application-specific implementation barriers, and the evolution of reported sensing performance.

Recent review articles have examined FBG sensing technologies from different perspectives, including sensor design, application-specific developments, and sensing performance. Representative examples are summarized in Table 1. Collectively, these reviews provide comprehensive insights into the design, development, and applications of FBG sensing technologies. However, comparatively less attention has been devoted to systematically evaluating deployment maturity as documented in the scientific literature across different application domains.

Although successful practical implementations of FBG sensing systems have been reported in different application domains, relatively few peer-reviewed studies describe their long-term operation under real service conditions. This review therefore evaluates deployment maturity using evidence reported in the scientific literature, with the aim of highlighting the gap between the rapid growth of academic research and the limited published evidence of sustained field operation.

This paper presents a three-dimensional analytical assessment of the current state of FBG sensing technology and seeks to address the following questions:

1. To what extent have FBG sensing systems progressed from laboratory-oriented research toward field and operational deployment?

2. How does deployment maturity differ across major application domains, including structural health monitoring, biomedical systems, industrial monitoring, chemical sensing, and harsh-environment applications?

3. How has the reported sensing performance of representative FBG-based systems evolved over the past five years?

4. Which factors continue to limit the transition of FBG sensing systems from laboratory validation to long-term operational deployment?

To answer these questions, the first dimension of the analysis provides a quantitative assessment of published studies and distinguishes between laboratory-oriented and field-oriented research. The second dimension examines practical applications of FBG sensing systems across different domains, with particular attention to their capabilities and limitations under real operating conditions. The third dimension evaluates the evolution of reported sensing performance using representative literature data.

The primary objective of this work is to evaluate the transition of FBG sensing systems from laboratory research to practical deployment. By combining publication statistics, deployment-maturity classification, application-domain analysis, and sensing-performance trends, the study identifies the main factors associated with deployment maturity and long-term field deployment as reported in the scientific literature.

## 2. Research Methodology

### 2.1. Data Source and Time Span

The Scopus database was used as a consistent and quantitatively reliable source of publications. It was selected because of its broad coverage of peer-reviewed literature in engineering, optics, and applied sciences, as well as its suitability for large-scale bibliometric analyses. Only peer-reviewed journal articles published in English were included to ensure consistency throughout the literature screening and comparative analysis.

The analysis was limited to publications indexed between 2021 and 2026. This period was chosen to capture recent developments in FBG sensing systems, particularly those related to practical deployment and real-world applications. Focusing on this time span makes it possible to evaluate both current research trends and the maturity of FBG sensing technologies.

### 2.2. Search Strategy

A structured keyword-based search strategy was used to identify relevant publications. The search was designed to capture studies related to FBG sensing systems regardless of application area or validation level. The primary search query was:

{“Fiber Bragg Grating” OR “FBG”}

The search was conducted across titles, abstracts, and keywords to provide the broadest possible coverage of relevant publications. This approach enabled an unbiased assessment of the distribution of studies across different maturity levels without restricting the dataset to specific experimental conditions or application contexts.

Only peer-reviewed journal articles published in English were considered. Following data retrieval, duplicate records and non-relevant studies were removed through manual screening to ensure consistency with the scope of FBG sensing and monitoring systems.

The literature search included all publications indexed in the Scopus database up to the date of data collection. Consequently, the records for 2026 represent publications available at the time of the search and should be interpreted as a partial year rather than a complete annual record.

### 2.3. Deployment Maturity Classification Framework

A structured classification framework was developed to provide a systematic and comparable assessment of the technological maturity of FBG sensing systems across different application domains. The framework extends the conventional binary distinction between laboratory and field studies by introducing a five-level deployment maturity scale.

Each study was classified according to a hierarchical decision process based on five evaluation criteria: (i) the testing environment; (ii) the type of validation or verification; (iii) the strength of evidence for field deployment; (iv) the duration of experimental evaluation; and (v) the level of system integration. The testing environment was considered the primary criterion for maturity assignment, followed by the type of validation and the strength of field evidence. Experimental duration and system integration were used as supporting criteria when additional clarification was required. This approach emphasizes deployment readiness rather than measurement performance alone and provides a consistent basis for comparing studies across different application domains. Each study was assigned to a single deployment maturity level based exclusively on the explicit evidence reported in the original publication. The five deployment maturity levels are defined as follows:

- **Level 1—Concept and/or Model.** The study presents theoretical concepts, numerical models, or simulation results without a functional prototype.
- **Level 2—Laboratory Prototype.** The study presents FBG sensing system prototypes that have been experimentally validated under controlled laboratory conditions. Validation focuses on sensitivity, accuracy, and calibration characteristics without considering the influence of real operating environments.
- **Level 3—Validated Testbed/Pilot Demonstration.** The study reports validation beyond standard laboratory conditions, for example, through pilot experiments, controlled testing on living subjects, or testbeds that reproduce realistic operating scenarios. System-level functionality is demonstrated, but long-term stability and operation under real environmental conditions have not been verified.
- **Level 4—Short-Term Field Deployment.** The sensing system has been deployed under real operating conditions and field data have been collected. However, the deployment remains short-term, project-specific, limited in scale, and does not provide evidence of sustained operation over time.
- **Level 5—Long-Term Operational Deployment.** The study demonstrates stable long-term operation of an FBG sensing system under real service conditions and provides evidence of reliability, robustness, maintenance feasibility, and integration into operational workflows over an extended period.

**Figure 1:**
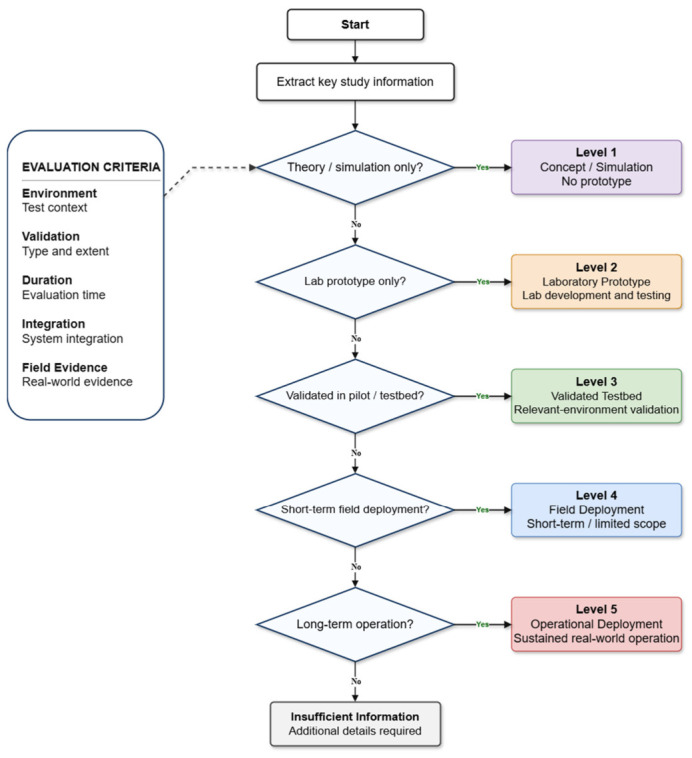
Decision-based framework for classifying the deployment maturity of FBG sensing studies, assigning studies across five levels from concept/simulation (Level 1) to long-term operational deployment under real service conditions (Level 5).

The transparency and reproducibility of the classification process were ensured through the hierarchical decision procedure illustrated in Figure 1. Classification was based exclusively on explicit evidence reported in the original publications, thereby minimizing subjective interpretation and improving the consistency of the maturity assessment across the reviewed dataset.

It is important to note that the proposed classification focuses specifically on deployment maturity and does not take the measurement performance of the systems into account. High sensitivity or accuracy achieved under laboratory conditions does not necessarily correspond to a higher level of technological maturity. This distinction is essential for identifying the gap between technological capability and practical deployment.

The proposed approach provides a quantitative and comparative assessment of the transition from laboratory research to field deployment and enables the identification of domain-specific barriers and factors that limit the progression of solutions toward higher maturity levels.

### 2.4. Dataset Construction and Classification Results

**Figure 2:**
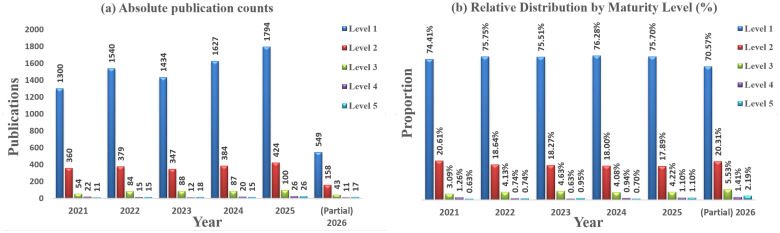
Temporal distribution of FBG sensing studies across five deployment maturity levels (2021–2026). Data for 2026 represent a partial year and include publications indexed in Scopus up to the literature search date. (**a**) Absolute number of publications by maturity level. (**b**) Relative distribution of publications expressed as normalized proportions (%).

A total of 10,960 publications published between 2021 and 2026 were identified and classified. The 2026 records correspond to publications indexed up to the literature search date and therefore represent a partial year. Figure 2 presents both the absolute number of publications and their relative distribution across the five deployment maturity levels between 2021 and 2026.

Figure 2a illustrates the overall increase in publication activity between 2021 and 2025, whereas Figure 2b shows that the relative distribution across deployment maturity levels remained largely unchanged throughout the study period.

The resulting distribution exhibits a pronounced imbalance at the lower maturity levels. Level 1 consistently dominates throughout the entire period, accounting for ~70–75% of all publications. This suggests that a large proportion of current research activity remains concentrated on conceptual, simulation-based, and laboratory-oriented developments. Level 2 represents the second largest category, with relatively stable proportions of ~17–20%, further highlighting the dominant role of laboratory-based validation within the current research landscape. The representation of higher levels of technological maturity is considerably lower. Level 3 accounts for a moderate share of publications, ranging from ~3% to 5%. Of greater significance, studies documenting real-world deployment are extremely scarce. Publications at Level 4 constitute only ~0.5–1.5% of the total, whereas those at Level 5 account for less than 2% across the entire study period. It is noteworthy that Levels 4 and 5 exhibit comparable proportions. However, the available data do not permit conclusions regarding the relative difficulty of transitions between individual maturity levels.

Consequently, the data presented indicate not only the predominance of early stages of development, but also the continued gap between research activity and the proven operational maturity of FBG sensor systems. Although a slight increase in higher maturity levels can be observed in recent years, it remains insufficient to produce a substantial change in the overall distribution.

### 2.5. Statistical Analysis and Discussion

Despite the substantial increase in the number of FBG-related publications, the overall maturity distribution remains remarkably stable. Most studies continued to be concentrated within Levels 1 and 2, while publications reporting field deployment represented only a small proportion of the dataset. This observation suggests that research activity is expanding primarily through the development of new sensing concepts and laboratory-scale demonstrations rather than through the translation of existing technologies into operational applications.

**Figure 3:**
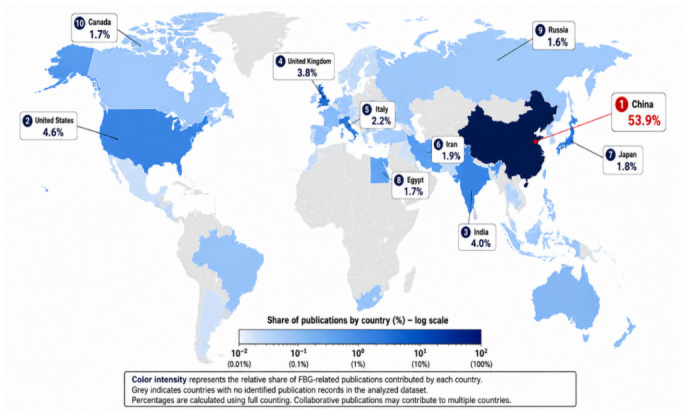
Global geographical distribution of FBG-related publications (2021–2026). Color intensity represents the relative publication share by country using a logarithmic scale.

The geographical distribution of publications, presented in Figure 3, further highlights the uneven nature of global research activity. China emerged as the dominant contributor, followed by the United States, India, the United Kingdom, and Italy. Research output was largely concentrated in countries with strong photonics capabilities, advanced manufacturing sectors, and established industrial research ecosystems.

When publication activity is considered together with deployment maturity, an additional pattern becomes apparent. Studies involving short-term field deployment or long-term operational deployment were more frequently reported in regions with substantial industrial engagement in infrastructure monitoring and sensing technologies. This suggests that technological deployment is influenced not only by scientific activity, but also by the presence of industrial demand, implementation opportunities, and supporting engineering infrastructure.

A comparison between lower-maturity studies (Levels 1–2) and field-oriented investigations (Levels 4–5) reveals a persistent deployment gap. In several years, conceptual and laboratory-based studies outnumbered field-deployment studies by more than an order of magnitude. Therefore, the growth of the literature does not necessarily correspond to an equivalent increase in literature-reported field deployment.

Overall, the results indicate that the current challenge facing FBG sensing technology is not the generation of new sensing concepts, but the progression of existing solutions toward reliable operation under practical conditions. The nature and severity of this challenge vary across application domains and are examined in the following sections.

## 3. Application Domains of FBG Sensors

The distribution of FBG sensing system applications across different domains was quantitatively analyzed to identify the dominant research and application areas. The analysis revealed a pronounced imbalance. Biomedical applications account for more than 30% of the publications, followed by chemical and biochemical sensing, which represent approximately 25%. Structural health monitoring, including transportation and underground infrastructure, accounts for about 15%, while industrial monitoring and sensing for harsh environments represent approximately 6% and 2% of the publications, respectively. A substantial proportion of the literature, approximately 20%, consists of general sensing studies or methodology-oriented research. This indicates the widespread presence of developments that are not associated with a specific application domain.

The distribution of publications across application domains provides a quantitative basis for evaluating the distribution of technological maturity levels within different fields. The reviewed studies were categorized according to their primary application domain because the intended application determines the operating conditions and practical challenges encountered by FBG sensing systems. Material selection and sensor structure were considered within each domain whenever they influenced sensing performance or practical deployment, rather than being treated as separate classification categories. Accordingly, five major application domains of FBG sensing systems were selected for detailed analysis to identify the practical limitations and system-level challenges that hinder the progression of these technologies toward higher maturity levels.

### 3.1. Biomedical Sensing Systems

Although biomedical sensing represents the largest share of the analyzed publication dataset, accounting for more than 30% of all studies, its deployment maturity remains lower than that observed in other application domains. For this analysis, a subset of 50 representative studies was selected to evaluate deployment maturity, integration requirements, and practical implementation challenges.

Biomedical applications of FBG sensing systems span physiological monitoring, rehabilitation, prosthetics, minimally invasive surgery, and biochemical diagnostics. Several studies combine FBG sensors with other optical sensing techniques to monitor multiple physiological or biochemical parameters within a single platform. For example, integrated optical systems incorporating FBG, interferometric, and plasmonic sensing elements have been proposed for simultaneous biomarker and temperature monitoring, enabling the concurrent detection of multiple clinically relevant variables [15].

Wearable and rehabilitation applications represent one of the most active areas of research. FBG sensors have been incorporated into smart textiles, gait-monitoring systems, and pressure-sensitive wearable devices for continuous physiological assessment. Flexible FBG-based pressure sensors have demonstrated sensitivities of up to 13.37 pm/kPa and have been applied to plantar-pressure monitoring for rehabilitation and movement assessment [16]. Human-subject studies have also demonstrated the simultaneous acquisition of respiratory, cardiac, and speech-related information using wearable FBG platforms, with reported speech-recognition accuracies exceeding 96% under controlled conditions [17].

Prosthetic systems represent a distinct biomedical application area for FBG sensing technologies. FBG sensor networks embedded within advanced prosthetic feet have been used to monitor strain distribution, loading conditions, and energy-transfer mechanisms during gait. Experimental studies have reported improvements in energy efficiency of up to 15.5% for optimized prosthetic designs, demonstrating the value of distributed sensing for biomechanical assessment and prosthesis development [18].

Minimally invasive and implantable sensing systems form a further group of biomedical applications. Hybrid FBG-Fabry-Pérot devices have been developed for simultaneous pressure and temperature monitoring in compact biocompatible structures. Reported pressure sensitivities exceed 200 pm/kPa while maintaining temperature-monitoring capability, making such devices suitable for internal physiological measurements [19]. In surgical applications, FBG-based instruments have been employed for tissue-stiffness assessment and force monitoring during endoscopic procedures. Recent studies have demonstrated in vivo use of such systems in surgical environments, confirming technical feasibility while also highlighting the challenges associated with clinical implementation [20].

Machine-learning methods are used for signal interpretation and parameter estimation in FBG-based biomedical systems. Applications include non-invasive glucose monitoring, multimodal physiological monitoring, and clinical decision-support systems [21,22].

The maturity classification shows that most of the biomedical FBG sensing systems remain within Levels 2 and 3. Level 1 studies are relatively uncommon and are mainly represented by conceptual biosensor designs and simulation-based investigations that have not yet progressed to experimental validation [23]. Level 2 studies primarily demonstrate technical feasibility under controlled laboratory conditions, whereas Level 3 studies include limited validation involving human subjects or semi-realistic operating scenarios [16,17,18]. Only a limited number of publications reach Level 4, where FBG sensing systems are evaluated under clinically relevant conditions, including rehabilitation monitoring and biochemical sensing using real samples [15,20,24]. No examples of sustained long-term operational deployment corresponding to Level 5 were identified within the analyzed dataset.

**Figure 4:**
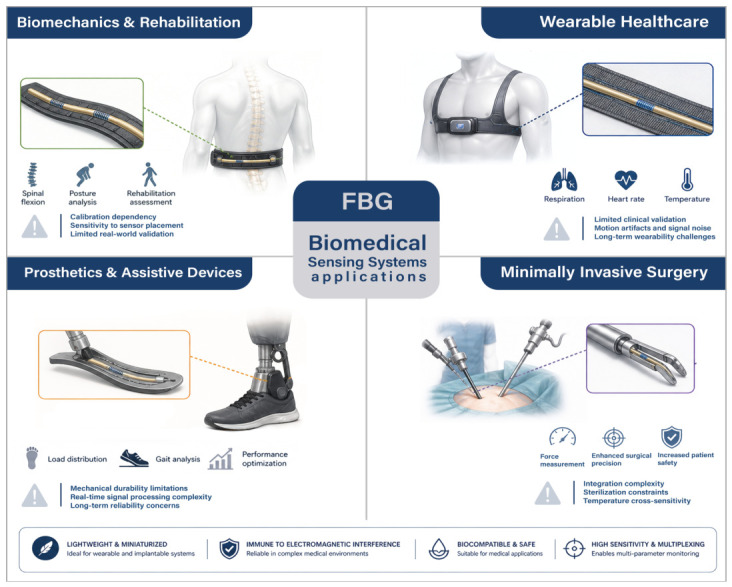
Original schematic illustration of representative biomedical applications and associated deployment challenges of FBG sensing systems across wearable healthcare, rehabilitation, prosthetics, and minimally invasive surgical systems, synthesized from representative published studies [25,26,27,28,29].

Biomedical FBG sensing systems are increasingly applied in wearable healthcare, rehabilitation, prosthetics, and minimally invasive clinical technologies, as illustrated in Figure 4.

Despite the diversity of biomedical applications, the barriers limiting deployment are mainly associated with clinical integration rather than with the basic ability of FBG sensors to detect physiological or biomechanical signals. Wearable and rehabilitation systems require validation on larger and more diverse patient cohorts, longer observation periods, and assessment of sensor stability during repeated daily use. Prosthetic applications additionally require robust embedding strategies, mechanical durability, and reliable strain transfer under cyclic loading.

For minimally invasive and implantable systems, the main constraints are more stringent. Practical implementation requires miniaturization, biocompatible packaging, sterilization compatibility, mechanical robustness, and safe integration with existing surgical or diagnostic instruments. These requirements make the transition from experimental prototypes to clinically usable devices more difficult than in many structural or industrial applications.

Table 2 summarizes representative application-specific limitations that arise during the transition of biomedical FBG sensing systems from controlled validation to clinically meaningful use.

Overall, biomedical FBG sensing demonstrates strong technical feasibility, but its deployment is constrained by application-specific requirements that are difficult to reproduce in laboratory experiments. Future progress in this domain therefore depends on clinically relevant validation protocols, long-term usability studies, and engineering solutions that ensure stable operation under clinically relevant conditions.

### 3.2. Chemical and Biochemical Sensing

Chemical and biochemical applications differ from conventional FBG sensing because the target analyte usually does not affect the grating directly. In contrast to strain, temperature, or pressure measurements, where the measurand can be converted into a Bragg-wavelength shift through the intrinsic response of the fiber, chemical and biochemical sensing typically requires an intermediate functional layer or transduction structure. Interaction between the analyte and this functional material may change its swelling state, refractive index, optical loss, or mechanical stress, and these changes are then transferred to the FBG as a measurable optical response.

**Figure 5:**
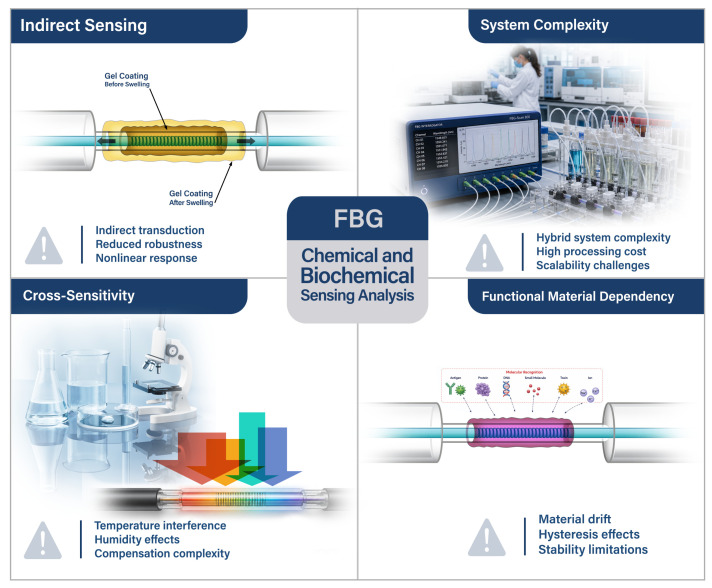
Conceptual illustration of the sensing mechanisms and literature-reported barriers in FBG-based chemical and biochemical sensing, including indirect transduction, cross-sensitivity, system complexity, and functional-material dependency [34,35,36,37,38].

This indirect transduction mechanism enables the detection of gases, pH, biochemical markers, humidity, corrosion-related processes, and other analytes. At the same time, it shifts the deployment problem from the grating alone to the stability and reproducibility of the complete sensing interface. As a result, the practical performance of FBG-based chemical and biochemical sensors depends strongly on functional-material stability, selectivity, reversibility, temperature cross-sensitivity, calibration procedures, and interrogation requirements, as summarized conceptually in Figure 5.

Although chemical and biochemical sensing accounts for a substantial share of FBG-related research activity, most of the reported systems remain confined to laboratory validation. This indicates a significant gap between the sensitivity demonstrated by functionalized FBG sensors and the practical maturity required for stable long-term operation in real environments.

An analysis of 41 studies showed that most of the chemical and biochemical FBG sensing systems remain at the laboratory-validation stage (Level 2), while only a limited number of studies report pilot-scale or field-oriented implementations (Levels 3–4). No evidence of long-term operational deployment (Level 5) was identified. Representative examples of deployment barriers identified across the analyzed studies are summarized in Table 3. The reported deployment maturity indicates the maturity level or maturity range at which the corresponding barrier was identified across the reviewed literature. These values summarize evidence across the application domain and do not represent the maturity classification of individual studies.

The practical limitations of this approach become evident in specific sensing systems, where the measured response is governed not only by the FBG itself but also by the properties of the functional transduction layer. For example, Pd- and PDMS-coated FBG sensors were used for hydrogen and methane monitoring in transformer oil over concentration ranges of 5–2146 ppm H_2_ and 3–1551 ppm CH_4_, respectively [45]. Similarly, a Michelson–FBG sensor developed for DMMP detection achieved a sensitivity of 86.44 dB/ppm with a detection limit of 0.1767 ppb [46]. Although these systems demonstrate high sensitivity, their response remains dependent on intermediate materials and transduction processes rather than on direct analyte interaction with the grating [62].

Material-related limitations are particularly evident in commonly used coating families. Pd- and Pd-alloy-based hydrogen sensors may suffer from phase-transition effects and slow response or recovery dynamics [39,41], whereas polymer-based pH and humidity sensors are affected by swelling, temperature-dependent behavior, and gradual material degradation [40,42]. Thus, in functionalized FBG sensors, long-term performance is often governed by the stability and reversibility of the coating rather than by the intrinsic properties of the grating itself.

Temperature sensitivity remains a persistent problem in chemical and biochemical FBG sensing. In many reported systems, thermally induced wavelength shifts are comparable to, or greater than, the spectral response produced by analyte interactions. Consequently, additional sensing elements or compensation procedures are often required. Reference gratings, cascaded FBG configurations, and sensitivity-matrix approaches are therefore commonly employed in multiparameter sensing systems [47,54,56,63].

To improve selectivity, many studies combine FBG sensors with interferometers, microfluidic devices, plasmonic structures, or spectroscopic techniques. Such architectures improve sensing performance but require more complex and signal-processing optical schemes. Methane-monitoring systems, for example, combine wavelength-modulation spectroscopy with FBG sensing and require multiple processing stages for signal extraction [56]. Similar approaches are reported in biochemical sensing platforms employing cascaded sensing elements and matrix-based demodulation methods [47,50].

Signal interrogation also remains an important practical consideration. In several advanced sensing systems, useful information is encoded in narrow spectral features or complex resonance patterns that require high-resolution interrogation and computational post-processing [51,52,53].

Existing pilot and field-oriented examples should therefore be interpreted as boundary cases that demonstrate deployment feasibility rather than full operational maturity. Methane-monitoring networks have been tested at eight sensing locations in an operating gas-processing facility [56], and battery-monitoring systems have been validated in commercial lithium-ion cells over more than 100 charge–discharge cycles [64]. These studies are important because they move beyond purely laboratory conditions; however, they still provide limited evidence regarding aging, recalibration, maintenance requirements, and stable performance during prolonged operation under representative conditions.

As the number of sensing points increases, the deployment problem shifts from the performance of individual sensors to the architecture of the entire monitoring system. Large-scale FBG networks require high-speed interrogation, stable optical power budgets, reliable channel management, and sufficient data-processing capacity [56]. Similar system-level constraints are observed in battery-monitoring applications, where sensor performance must be balanced against implementation cost, packaging complexity, and the practical feasibility of integration into commercial cells [57].

Taken together, these observations indicate that the main limitation of chemical and biochemical FBG sensing is the transition from sensitive laboratory devices to stable deployable measurement systems. Reported sensitivities and detection limits confirm that the optical response is often sufficient for analyte detection; however, practical deployment depends on whether the functional layer, calibration model, temperature-compensation strategy, interrogation scheme, and overall system architecture remain stable under realistic operating conditions. Therefore, further progress in this field should be evaluated not only by lower detection limits or higher sensitivity, but also by reproducibility, drift, selectivity, maintenance requirements, and long-term validation in representative environments.

### 3.3. Structural Health Monitoring Applications

Structural health monitoring is a highly compatible application domain for FBG sensing because its primary measurands, including strain, temperature, vibration, displacement, and pressure, can be directly converted into Bragg-wavelength shifts. This makes FBG sensors well suited for bridges, concrete structures, transportation infrastructure, underground facilities, pipelines, and other civil-engineering assets. For this analysis, 90 representative studies were examined to assess measurement performance, deployment maturity, integration conditions, and system-level constraints.

**Figure 6:**
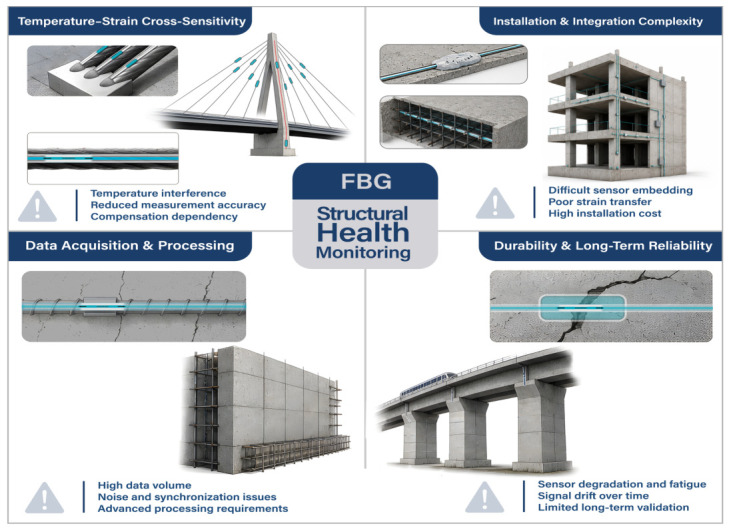
Representative FBG-based structural health monitoring (SHM) applications in civil infrastructure and the associated operational challenges reported in the literature [2,65,66,67,68,69].

In SHM applications, deployment maturity should be evaluated at the level of the complete monitoring system rather than at the level of the grating alone. A practical FBG-based SHM platform includes sensor installation, strain-transfer interfaces, environmental protection, optical interrogation, data transmission, signal interpretation, and maintenance procedures, as illustrated in Figure 6. Therefore, field performance depends not only on the accuracy of wavelength-shift measurements, but also on installation quality, calibration stability, data continuity, and the ability to interpret sensor responses over long monitoring periods.

The following subsections examine four representative SHM areas: bridge structures, concrete buildings and structural elements, transportation infrastructure, and underground or large-scale infrastructure. This structure makes it possible to distinguish between domains where FBG systems have already reached field deployment and domains where validation remains mainly laboratory- or pilot-oriented.

#### 3.3.1. Bridge Structures

A number of studies dedicated to bridge monitoring via the FBG technology report deployments under real operating conditions, including long-span suspension bridges, post-tensioned concrete bridges, railway bridges, and bridge weigh-in-motion systems [70,71,72,73,74,75,76].

The reviewed studies demonstrate that FBG sensing systems are capable of providing accurate measurements under field conditions. For example, high-strength steel-wire FBG sensors installed on an operating suspension bridge enabled real-time monitoring of cable tension during a multi-month construction period, with cumulative force-identification errors below 5% [72]. In another study, static proof-load testing of a six-span post-tensioned bridge showed agreement between measured and simulated responses, with strain and deflection differences generally not exceeding ~10% [73].

Many reported implementations combine FBG sensors with finite-element models, machine-learning tools, and other data-processing approaches to improve data interpretation and compensate for environmental influences. Temperature remains one of the main sources of measurement uncertainty, since thermally induced wavelength shifts can be comparable to, or even greater than, those produced by structural loading. For this reason, most practical systems require additional compensation methods, reference sensors, or advanced signal-processing techniques to distinguish thermal effects from mechanical responses [71,74].

Bridge applications provide some of the strongest evidence of real-world deployment among FBG sensing technologies. Their remaining limitations are mainly associated with transition from project-specific monitoring campaigns to lifecycle-scale bridge management. This requires stable temperature compensation, standardized installation procedures, reliable interpretation of long-term data, and integration of FBG measurements into routine inspection and maintenance workflows.

#### 3.3.2. Concrete Buildings and Structural Elements

Applications of FBG sensing systems in concrete structures are mainly associated with corrosion monitoring, early-age shrinkage assessment, crack development, and the evaluation of stress evolution within reinforced concrete elements [60,77,78,79,80,81,82,83,84,85]. Most of the reviewed studies were carried out under laboratory or semi-controlled conditions, whereas reports involving long-term monitoring of real structures remain relatively limited. Several studies demonstrated the capability of embedded fiber-optic sensors to monitor deterioration processes within concrete structures. For example, embedded FBG sensors were successfully used to monitor corrosion-induced expansion in reinforced concrete specimens and to correlate strain development with different stages of corrosion progression [77]. In another study, FBG sensors embedded at different locations inside concrete specimens were used to monitor alkali–silica reaction (ASR) over a period of 365 days. The measured expansion reached 0.114% in cylindrical specimens and 0.053% in prisms, while sensors embedded directly within coarse aggregate particles detected local expansions as low as 0.015%, providing insight into the early stages of internal deterioration [84]. Distributed fiber-optic sensing systems continuously monitored early-age concrete deformation during the first 24 h after casting, allowing the identification of shrinkage-related strain development before visible cracking occurred [85].

FBG sensing systems have demonstrated the ability to monitor a wide range of deterioration processes in concrete structures with high accuracy. Nevertheless, long-term performance depends on reliable sensor integration and durability under harsh structural environments. Most of the reported studies focus on laboratory specimens or individual structural elements, whereas evidence from sustained monitoring of full-scale infrastructure remains relatively scarce.

#### 3.3.3. Transportation Infrastructure

Transportation infrastructure is among the most representative application areas in which FBG sensing systems have been validated under real operating conditions. Reported studies cover railway transition zones, pavement monitoring systems, highway subgrades, and intelligent transportation infrastructure [67,86,87,88,89,90,91,92,93].

Several studies demonstrated the feasibility of long-term monitoring under operational traffic loading. In a railway transition zone between ballasted and slab track, an FBG-based monitoring system was installed on an operating railway line in northern Sweden using four instrumented measurement clusters. The system provided continuous measurements for the assessment of track-bed degradation and was used for calibration of numerical models under real service conditions [67]. The same study noted that more than 50% of the investigated bridge-transition zones exhibited differential settlement, with an average defect length of 5.2 m and an average depth of 33 mm [67].

Field applications have also been reported for road infrastructure. An ultra-weak FBG sensing array was used for the detection of underground voids and sinkholes beneath operating expressways, where the proposed methodology was validated using 101 field data samples without interrupting traffic flow [89]. In another study, a pavement-monitoring system based on embedded FBG sensors collected data continuously over a four-month period and applied automated anomaly-detection algorithms to identify abnormal structural behavior under real traffic conditions [86].

At the same time, most of the reported implementations remain associated with specific infrastructure sections or pilot projects rather than network-scale deployment. Several monitoring systems rely on advanced data-fusion, machine-learning, or anomaly-detection algorithms to process large volumes of sensor data and improve interpretation reliability [86,88,93]. For example, intelligent pavement systems combining FBG sensors with distributed monitoring architectures achieved vehicle-classification accuracies of 95.7% and rut-depth measurement resolutions of ±1.5 mm on a 1.2 km highway testbed [92].

For transportation infrastructure, the deployment problem is strongly related to durability under repeated traffic loading and to the management of large monitoring datasets. Embedded sensors must remain stable under cyclic mechanical loading, moisture exposure, temperature fluctuations, and pavement or track degradation [87,91,94], while continuous monitoring requires reliable data storage, automated quality control, and robust anomaly-detection procedures.

#### 3.3.4. Underground and Large-Scale Infrastructure

Underground and large-scale infrastructure applications also belong to the number of areas in which FBG sensing systems have progressed beyond laboratory validation. Reported applications include highway subgrades, diaphragm walls, subway stations, dike monitoring systems, and geotechnical support structures operating under real service conditions [95,96,97,98,99]. For example, an ultra-weak FBG sensing array was deployed during the expansion and renovation of a highway in Fujian Province, enabling distributed monitoring of strain and temperature over the entire subgrade structure [95]. In another study, distributed fiber-optic monitoring was implemented during the assembly of a prefabricated subway station in Shenzhen with a total length of approximately 212 m, where measured strains ranged from −260 με to 393.8 με during different construction stages [96]. Field-scale deployment was reported for water-level monitoring, where four FBG pressure sensors were installed and tested on a full-scale dike in the Netherlands [97].

Several limitations remain for practical implementation. As monitoring distances and the number of sensing points increase, the requirements for data acquisition, storage, and processing become considerably more demanding. Long-term operation requires stable calibration procedures and reliable compensation of environmental influences. In addition, many systems employ auxiliary sensing elements, mechanical transducers, or hybrid sensing architectures to improve measurement performance [97,98,99]. While these approaches expand sensing capabilities, they also increase system complexity and affect calibration stability.

Machine-learning-based approaches are increasingly used for data interpretation and condition assessment [98,99]. However, their performance remains dependent on the availability and quality of training data, while the transfer of trained models between different infrastructure systems and operating conditions continues to require further validation.

These examples show that FBG sensing systems can be implemented in large-scale underground and geotechnical infrastructure, including subgrades, subway stations, dikes, and retaining structures. In this domain, deployment maturity is mainly determined by scale-related factors: long monitoring distances, large numbers of sensing points, changing construction stages, variable boundary conditions, and the need for stable data interpretation over time. Further progress therefore depends on monitoring architectures that remain calibrated, interpretable, and maintainable throughout extended operation under real service conditions.

#### 3.3.5. Cross-Domain Synthesis

The cross-domain comparison presented in Table 4 shows that the deployment barriers in SHM applications differ in their technical form but converge at the system level. Bridge monitoring is limited mainly by lifecycle validation and temperature compensation; concrete structures by embedding, strain-transfer stability, and durability; transportation infrastructure by installation robustness, cyclic loading, and data continuity; and underground or geotechnical systems by scale, calibration drift, and data-management complexity. These differences indicate that the main obstacle to wider deployment is not the sensing capability of FBG sensors themselves, but the integration of sensing elements, interrogation systems, compensation procedures, and data-interpretation methods into reliable long-term monitoring platforms.

Thus, SHM is among the most mature FBG application domains, but its maturity is uneven. Bridge and large-scale infrastructure monitoring provide the strongest evidence of field deployment, whereas concrete and advanced optical sensing systems remain more strongly concentrated at laboratory or prototype levels.

#### 3.3.6. Critical Research Gap and Deployment Insight

The reviewed SHM studies indicate that FBG sensing has already reached a level of measurement capability sufficient for many structural-monitoring tasks. Therefore, the critical research gap lies not in proving that FBG sensors can detect structural responses, but in demonstrating that complete FBG-based SHM systems can operate reliably over long periods as part of real infrastructure-management workflows.

Most of the published studies still evaluate FBG systems primarily using sensor-level or experiment-level metrics, such as sensitivity, resolution, short-term repeatability, agreement with numerical models, or response under controlled loading. Much less attention is given to service-level criteria, including installation repeatability, strain-transfer stability, calibration drift, sensor survivability, data loss, environmental degradation, maintenance procedures, and the long-term interpretability of monitoring data. As a result, even technically successful demonstrations often provide limited evidence of lifecycle-scale reliability.

As the scale of the monitored structure increases, the same deployment barriers become increasingly important: temperature compensation, bonding or embedding quality, environmental protection, data continuity, dependence on numerical or data-driven interpretation models, and increasing complexity of the interrogation architecture. These issues are especially important when FBG monitoring is expected not only to record structural responses, but also to support operational decision-making.

Thus, the main deployment challenge in SHM is the transition from validated sensing elements to robust monitoring platforms. Future research should therefore place greater emphasis on long-duration field trials, standardized validation protocols, scalable interrogation architectures, uncertainty assessment, and maintenance-oriented system design. Such work is necessary to move FBG-based SHM from project-specific demonstrations toward routine operational use in civil and industrial infrastructure.

### 3.4. Industrial Process Monitoring and Smart Manufacturing Systems

**Figure 7:**
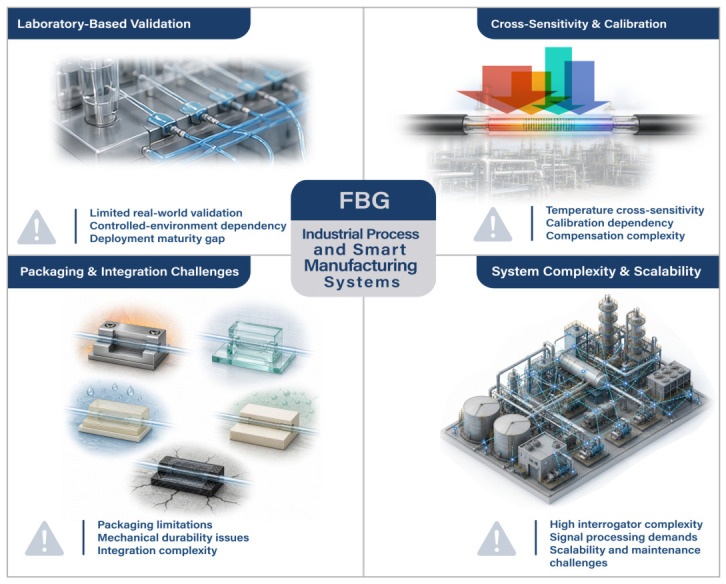
Overview of the principal deployment barriers reported in the literature that may hinder the transition of FBG sensing systems from laboratory validation to field deployment, including cross-sensitivity, packaging and integration challenges, and system complexity [57,112,113,114].

Industrial monitoring and smart manufacturing represent rapidly growing application areas of FBG sensing technology. Reported applications include gas sensing [103], injection-mold temperature monitoring [104], vibration-based fault detection [105], IoT-assisted temperature sensing [106], railway monitoring [107], pipeline-flow monitoring [108], transformer diagnostics [109], and sensing systems integrated into manufacturing processes [110]. The principal factors influencing deployment are summarized in Figure 7.

A review of 35 studies shows that the majority of the industrial FBG sensing systems remain at the laboratory-validation stage, while only a limited number have progressed to pilot testing or short-term field deployment. Examples include injection-molding systems in which FBG sensors were embedded directly into metallic molds and used to monitor temperature at 15 sensing locations during the manufacturing cycle [104]. Railway, pipeline, and industrial monitoring systems have also been reported [107,108], although validation is commonly performed on dedicated test facilities or pilot-scale installations rather than under long-term operating conditions.

Several studies demonstrate successful pilot-scale operation or short-term field deployment in industrial or semi-industrial environments. Methane-monitoring systems have been tested under field conditions [56], while hybrid FBG–Fabry–Perot sensing platforms were validated on a 220 kV power transformer for simultaneous pressure and temperature monitoring [109]. In smart-manufacturing applications, classification accuracies of 99.27% were reported for identifying operational states in fine-blanking processes [110]. However, most of the reported deployments remain limited in duration. For example, plant-monitoring systems were typically evaluated over periods of 7–15 days [111], and no evidence of continuous long-term operation corresponding to Level 5 deployment was identified.

Representative examples of deployment barriers identified across the analyzed studies are summarized in Table 5.

Temperature influence remains one of the most frequently reported practical difficulties in industrial monitoring applications. Dual-FBG configurations, cascaded sensing structures, and hybrid sensing architectures are commonly employed to separate temperature effects from the target measurand [115,116,117,118]. Their performance depends on calibration quality and the long-term stability of compensation procedures.

The integration of FBG sensors into industrial equipment introduces additional engineering constraints. Sensors have been embedded into metallic molds [104], metallic structures [119], composite components [120], and industrial safety systems [121]. Under such conditions, measurement quality depends not only on the grating itself but also on strain-transfer efficiency, bonding quality, thermal-expansion mismatch, and protective packaging. For metallic and welded industrial components, the reliability of embedded or surface-mounted sensing systems is also affected by the microstructural heterogeneity, residual stresses, and mechanical-property variations introduced during laser welding, repair, or joining of dissimilar materials [122].

A considerable number of studies focus on interrogation systems, multiplexing architectures, and signal-processing methods. Recent interrogation platforms achieved channel bandwidths of approximately 0.24 nm, enabling high spectral resolution, together with inter-channel crosstalk levels near −45 dB [123]. Image-sensor-assisted interrogation combined with convolutional neural networks achieved wavelength resolutions of approximately ±1 pm with prediction accuracies approaching 98% [124]. These interrogation systems provide high measurement performance; however, they require dedicated hardware and increasingly complex signal-processing procedures.

**Table 5:** Key Barriers Limiting the Practical Deployment of FBG-Based Sensing Systems.

Application Context	Validation Environment	Deployment Maturity	Key Limitation	Deployment Impact	Refs.
Industrial and structural monitoring systems (SHM, pipelines, machinery)	Predominantly laboratory/controlled environments	2–3	Limited field validation	Performance not verified under real operating conditions	[103,104,105,106,107]
Field-deployable sensing systems (industrial, environmental, infrastructure)	Partial field validation/short-term deployment	3–4	Lack of long-term reliability	No evidence of long-term stability, drift, or aging behavior	[41,90,91,92]
Multi-parameter sensing systems (temperature–strain–pressure)	Laboratory validation	2–3	Cross-sensitivity and calibration dependency	Measurement affected by temperature and multi-parameter coupling	[115,116,117]
Embedded and material-integrated sensing systems	Laboratory/material-level validation	2–3	Packaging limitations	Reliability depends on embedding, bonding, and environmental protection	[119,120,121]
Optical sensing networks and interrogation architectures	Laboratory/simulation-based validation	1–2	Interrogation complexity	High cost and complexity of interrogation and signal processing systems	[123,124,125,126,127,128]
Chemical and functionalized sensing systems	Laboratory/controlled environments	2	Material dependency	Performance influenced by functional materials and intermediate transduction layers	[94,98,104]
Large-scale and distributed sensing systems	Laboratory/pilot-scale setups	2–3	Scalability constraints	Difficult integration into large-scale systems and distributed infrastructures	[89,91,97,105]

In process-monitoring systems, measurement performance often depends on auxiliary sensing elements, transduction structures, bonding layers, and protective packaging [116,121]. Consequently, long-term stability is influenced by the aging behavior of these components, their mechanical coupling with the monitored equipment, and their resistance to repeated process cycles.

In industrial monitoring, the deployment problem is strongly linked to the conditions of continuous production rather than to sensing performance alone. FBG systems must remain calibrated during thermal cycling, vibration, mechanical loading, and process-related disturbances, while their installation should not interrupt the technological workflow or increase equipment downtime. Sensor packaging, bonding, and embedding strategies therefore need to be compatible with industrial maintenance procedures, replacement cycles, and safety requirements. From this perspective, wider adoption of FBG sensing in smart manufacturing depends on whether the complete monitoring system can provide stable data with acceptable installation complexity, maintenance effort, and lifecycle cost.

### 3.5. Harsh Environment Sensing

Harsh-environment sensing represents a distinct deployment challenge for FBG technology because the sensing element must operate under conditions that simultaneously affect the optical fiber, protective packaging, mechanical coupling, and interrogation stability. Unlike many laboratory-oriented sensing studies, harsh-environment applications require the complete sensing system to remain functional under elevated temperature, high pressure, radiation exposure, cryogenic conditions, vibration, humidity, or chemically aggressive media.

**Figure 8:**
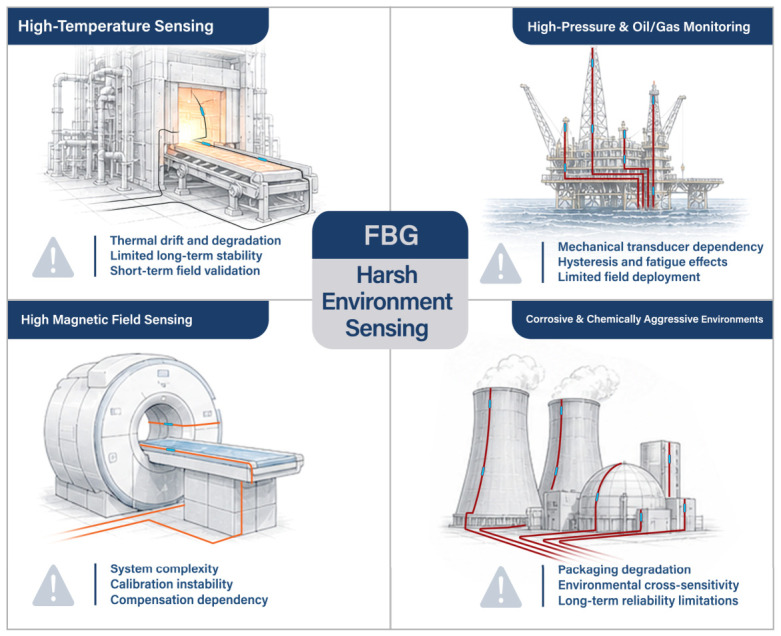
Representative FBG sensing applications in harsh environments and the corresponding operational challenges associated with high-temperature, high-pressure, high-magnetic-field, and chemically aggressive conditions [129,130,131,132,133].

FBG sensors are attractive for such applications because of their electromagnetic immunity, small size, multiplexing capability, and compatibility with remote optical interrogation. Reported studies include high-temperature industrial monitoring, oil-and-gas pressure sensing, cryogenic and superconducting systems, radiation-exposed facilities, dynamic mechanical measurements, and chemically aggressive environments. The main factors affecting deployment in these conditions are summarized in Figure 8.

In this domain, deployment maturity is determined not only by whether the FBG can detect the target measurand, but also by whether the surrounding engineering solution can preserve calibration, strain transfer, optical integrity, and environmental protection over time. Sensor packaging, material compatibility, sealing, thermal aging, mechanical stability, and compensation procedures therefore become central factors in assessing practical readiness.

A total of 80 studies were analyzed to evaluate deployment maturity and practical implementation barriers of FBG sensing systems in harsh environments. The reviewed publications cover high-temperature industrial systems, high-pressure oil-and-gas sensing, structural and strain-based monitoring, dynamic and multiparameter sensing, cryogenic and radiation environments, and chemically aggressive media.

#### 3.5.1. High-Temperature Industrial Systems

High-temperature applications constitute one of the most developed areas of FBG sensing. Reported studies include temperature monitoring of bottom-anode pins in a 165-ton electric arc furnace during a two-month industrial campaign [134], thermal profiling of submerged entry nozzles using sapphire FBGs operating up to 1600 °C with stability tests lasting 40 h [135], and waveguide-assisted femtosecond-laser-written FBG structures tested at 1000 °C for 24 h [136]. These results demonstrate that FBG sensors can operate under temperature conditions that are difficult for many conventional sensing technologies.

At the same time, the majority of the available studies remain limited to laboratory experiments, calibration procedures, or relatively short industrial trials. Even in applications where field deployment has been demonstrated, the reported observation periods range from several hours to a few months [134,135]. Information on thermal aging, packaging degradation, calibration stability, and durability under repeated thermal cycling remains limited. Therefore, the main difficulties are associated with long-term operation and reliability rather than with temperature measurement capability itself.

#### 3.5.2. High-Pressure and Oil and Gas Sensing

Pressure and temperature monitoring in oil and gas wells is one of the most demanding applications of fiber-optic sensing. Reported studies include FBG- and FPI-based systems designed for simultaneous pressure and temperature measurements under elevated pressure and temperature conditions. Experimental demonstrations have been reported up to 50 MPa and 150 °C using all-metal FBG sensor structures intended for long-term monitoring in oil and gas wells [137], while diaphragm-based FPI–FBG sensors have been tested up to 80 MPa and 150 °C and proposed for deployment in wells with depths reaching 3500 m [138].

The reported sensing systems employ pressure-sensitive mechanical structures, including thin-walled cylinders, metal diaphragms, and hybrid FPI–FBG configurations. As a result, measurement performance depends not only on the optical sensing element but also on the stability of the mechanical structure and strain-transfer mechanism. Several studies discuss creep, thermal effects, sealing, material selection, and temperature–pressure cross-sensitivity, requiring temperature compensation and repeated calibration procedures [137,138].

The available results confirm the feasibility of pressure measurements under conditions representative of oil and gas wells. At the same time, most of the reported studies are based on laboratory calibration and controlled testing environments. Information on long-term operation under real production conditions remains limited, particularly with respect to aging, calibration stability, and long-term reliability.

#### 3.5.3. Structural and Strain-Based Sensing

Structural monitoring applications include composite structures, industrial pipelines, subsea systems, and reinforced components [139,140,141]. An optical-fiber-integrated steel–FRP composite bar was reported for simultaneous strain and damage monitoring, with strain measurements up to 13,200 με and tensile strengths exceeding 800 MPa [139]. Corrosion monitoring of hydrogen-fluoride transportation pipelines was demonstrated using four FBG sensing points installed on a stainless-steel pipe during a 10 h accelerated-corrosion test. The developed model predicted relative corrosion depth with an error below 10% [140]. Embedded strain- and humidity-sensitive FBG sensors were integrated into watertight connectors and evaluated under high-pressure testing conditions for the detection of structural damage and sealing degradation [141]. The proposed approach was validated in laboratory pressure tests rather than during long-term marine operation.

Most of the relevant studies remain based on laboratory experiments, accelerated aging procedures, pressure-vessel testing, or dedicated test facilities. Information on long-term operation under real service conditions remains limited, particularly for subsea and corrosion-monitoring applications.

#### 3.5.4. Dynamic and Multiparameter Sensing

Blast-wave strain measurements have been reported using FBG sensors embedded in polypropylene and polytetrafluoroethylene (PTFE) specimens and tested inside a vertical shock tube [142]. For polypropylene specimens, maximum compressive strains of 445.3, 555.3, and 637.3 με were recorded under different blast-wave conditions, corresponding to strain rates of 128.53, 164.05, and 208.84 s^−1^, respectively [142].

High-temperature vibration monitoring was investigated using a dual-FBG sensor designed for simultaneous vibration and temperature measurements [130]. The sensor operated between 25 °C and 1100 °C, with a characteristic frequency of 2100 Hz and a vibration sensitivity of 0.286 pm/g in the range of 1–50 g after temperature compensation [130].

A cascaded Fabry–Perot–FBG structure was proposed for simultaneous measurement of temperature, acoustic signals, and vibration [143]. The sensor operated at temperatures up to 500 °C, with a sound-pressure sensitivity of 0.033 mV/Pa, a vibration sensitivity of 0.288 pm/g, an acoustic frequency range of 100 Hz–10 kHz, and a vibration frequency range of 10 Hz–4 kHz [143].

Most of the reported systems require temperature compensation, dedicated interrogation equipment, or combined sensing structures. Measurement performance depends on the stability of the interrogation system, calibration procedures, and signal-processing methods used during operation.

#### 3.5.5. Cryogenic and Radiation Environments

Cryogenic applications include quench detection in superconducting systems, temperature measurements at liquid-helium temperatures, and strain monitoring under cryogenic loading conditions [144,145,146]. In superconducting REBCO tapes, FBG sensors were investigated for quench detection in liquid nitrogen, where the response time of the FBG sensor was shorter than that of conventional voltage taps when the hotspot was located within 10 mm of the grating position [144].

Strain measurements at liquid-helium temperature (5 K) have also been reported using metallized FBG sensors. Cyclic loading tests up to 1000 N and overload tests up to 3000 N showed stable operation in the elastic region up to approximately 2500 N, with a strain sensitivity exceeding 1.26 pm/N and a hysteresis error below 0.1 nm [145].

Embedded FBG arrays have also been used for real-time strain monitoring in electrical penetration assemblies intended for nuclear applications, enabling continuous measurements during thermal cycling and thermo-mechanical loading processes [146].

Radiation-related studies have investigated the response of optical-fiber gratings to neutron irradiation [147]. UV-written and femtosecond-laser-inscribed FBGs were exposed to neutron fluences of approximately 8.6 × 10^15^ n/cm^2^, while long-period gratings were tested up to about 3.4 × 10^15^ n/cm^2^ [147]. The investigated gratings remained operational throughout the irradiation experiments.

Published information on long-term operation in large-scale superconducting installations and nuclear facilities remains limited.

#### 3.5.6. Chemically Aggressive Environments

Chemically aggressive environments include marine structures, sewer infrastructure, and gas-monitoring systems operating in the presence of reactive gases [46,148,149].

In marine applications, FBG sensors have been incorporated into epoxy-based coatings for monitoring biofilm formation and corrosion-related processes on carbon-steel surfaces exposed to seawater [149]. The recorded wavelength changes were associated with biofilm development and changes within the coating system. Gravimetric analysis conducted over 14 days showed lower weight loss in treated samples compared with sterile seawater controls. Electrochemical measurements indicated a maximum corrosion-inhibition efficiency of 81.97% on the second day, while electrochemical impedance spectroscopy showed maximum biofilm stability on the twelfth day [149].

Sewer infrastructure represents another challenging operating environment because of high humidity, condensation, and biologically induced corrosion. Optical-fiber temperature and humidity sensors have been investigated for wastewater systems, where relative humidity is typically above 90% [148]. The reported measurements were used to determine dew-point conditions and detect surface condensation associated with the initiation of corrosion processes in sewer pipelines [148].

Gas monitoring has also been investigated using boron-nitride-nanotube-coated (BNNT-coated) FBG structures [46]. The sensors were tested in ammonia, hydrogen chloride, and bromine atmospheres. Gas exposure produced reversible changes in reflected optical power, while the Bragg wavelength remained essentially unchanged [46]. After removal from the gas environment, the sensor response recovered within a short period, allowing repeated measurements. The temperature sensitivity of the FBG was preserved after BNNT deposition [46].

The reviewed studies indicate that operation in chemically aggressive environments is usually achieved using protective coatings, encapsulation layers, or functional surface materials. Under such conditions, measurement stability depends not only on the FBG itself but also on the long-term behavior of the surrounding materials during exposure to humidity, corrosive media, and chemically reactive substances.

#### 3.5.7. Synthesis of Limitations

The reviewed harsh-environment studies show that the main deployment barriers are concentrated at the engineering-system level. In most cases, the limiting factors are not associated with the intrinsic sensitivity of the FBG itself, but with the ability of the complete sensor assembly to preserve calibration, optical integrity, mechanical coupling, and environmental protection under prolonged exposure to severe operating conditions.

Practical implementation commonly requires additional engineering solutions, including protective packaging, pressure-resistant housings, high-temperature coatings, sealing elements, temperature-compensation procedures, hybrid interrogation schemes, and mechanically stable transduction structures. Representative examples include gas-monitoring systems that combine FBG sensing with wavelength-modulation spectroscopy [56], high-temperature furnace and boiler applications requiring dedicated thermal protection and packaging [135,150], and downhole or seismic oil-and-gas sensing systems requiring mechanically robust and environmentally protected sensor assemblies [151].

These examples show that successful operation has been demonstrated in several industrial, field-related, and harsh-environment test conditions. However, evidence of long-term operational deployment remains limited. For this reason, the key unresolved issues are the durability of packaging materials, calibration stability under combined environmental loads, reproducibility of sensor installation, long-term behavior of transduction structures, and serviceability of the complete monitoring system.

The principal limitations identified across the reviewed harsh-environment applications are summarized in Table 6.

Overall, FBG sensing technology has demonstrated clear feasibility for measurements in harsh environments. However, the transition from successful demonstrations to long-term operational deployment depends on qualification of the complete sensing system rather than on the grating alone. Future work in this area should therefore focus on long-duration aging tests, standardized packaging validation, calibration stability under combined loads, and maintenance-oriented sensor-system design.

## 4. Deployment Maturity Gap and Performance Stabilization in FBG Sensing Systems

The maturity distribution reported in Section 2 provides the baseline for comparing deployment progress with sensing-performance evolution. Instead of indicating a gradual shift toward operational use, the classification results show a persistent concentration of FBG studies at the early development stages, with Levels 1 and 2 together accounting for 93.94% of the dataset. In contrast, publications providing evidence of field or long-term operational deployment remain only a small fraction of the total dataset. This imbalance is important for the present analysis because it shows that improvements in sensing performance should not be interpreted as direct evidence of deployment maturity.

The distribution of maturity levels remained relatively stable during the period from 2021 to 2025. The proportion of Level 1 studies remained within the range of approximately 74–76%, while variations in the remaining categories were comparatively small. Although a higher proportion of advanced maturity levels appears in 2026, the available data for this year are incomplete and should be interpreted with caution.

To compare technology maturity with sensing performance, representative studies published between 2015 and 2026 were examined. The results are summarized in Table 7.

Table 7 shows that the reported temperature sensitivity of FBG-based sensors has remained within a relatively narrow interval, typically between approximately 12.6 and 18.1 pm/°C. Early studies based on regenerated fiber Bragg gratings reported sensitivities close to 15 pm/°C and established the basis for high-temperature sensing applications. Subsequent work investigated temperature-dependent behavior, femtosecond-inscribed gratings, fiber-laser interrogation methods, and various packaging approaches. Despite these developments, the reported sensitivity values remained within a comparatively narrow range.

From 2019 onward, many studies focused on interrogation techniques, packaging solutions, measurement stability, and operation under specific environmental conditions. Recent publications have also investigated distributed sensing, protective coatings, and multi-parameter measurement configurations. These developments improved practical performance and broadened the range of operating conditions, while the reported temperature sensitivity changed only slightly.

The data summarized in Table 7 indicate that recent developments have been associated primarily with interrogation methods, packaging approaches, environmental adaptation, and measurement stability. At the same time, the maturity-level analysis shows that field-deployed systems represent only a small proportion of the available literature. Information on long-term operation under practical conditions therefore remains considerably more limited than information obtained from laboratory investigations.

Taken together, the maturity classification and performance-trend analysis indicate that the lab-to-field gap in FBG sensing cannot be explained by insufficient sensing performance alone. Reported temperature sensitivities have remained within a relatively stable range, whereas research activity continues to be concentrated at conceptual and laboratory-validation levels. This suggests that recent progress has been driven mainly by interrogation methods, packaging approaches, compensation strategies, environmental adaptation, and measurement stability rather than by a fundamental change in intrinsic sensing performance.

Therefore, deployment maturity should be interpreted as a system-level property. The decisive factors are long-term calibration stability, robustness of packaging and transduction structures, integration into operational workflows, scalability of interrogation architectures, and evidence of sustained use under real service conditions. These factors differ across application domains and form the basis for the following discussion.

## 5. Discussion

The results presented in the previous sections show that the development of FBG sensing systems differs considerably between application domains. Structural health monitoring and several harsh-environment applications include examples of short-term field deployment and isolated long-term operational deployment, whereas biomedical, chemical, and several concrete-structure or prototype-oriented infrastructure applications remain dominated by laboratory validation and pilot-scale demonstrations.

One of the factors influencing this difference is the relationship between the measured parameter and the sensing mechanism of the FBG. Parameters such as strain, temperature, and pressure directly affect the Bragg wavelength and can therefore be measured using relatively simple sensing configurations. In contrast, chemical, biochemical, and some biomedical measurements usually require coatings, transducers, or additional optical structures to convert the target parameter into a measurable optical response. These additional elements increase calibration requirements and introduce additional sources of uncertainty.

Practical implementation is also influenced by the ease of sensor integration. In infrastructure, energy, and aerospace applications, FBG sensors can often be embedded directly into structural components such as pipelines, cables, composite materials, and pressure vessels. In other applications, including concrete structures, biological tissues, and chemically aggressive environments, installation and long-term operation are generally more complicated because of material compatibility, coating stability, environmental effects, and maintenance requirements.

A similar difference can be observed between laboratory validation and operation under practical conditions. Laboratory experiments are typically performed under controlled conditions, whereas field operation involves temperature variations, environmental influences, installation variability, and long-term aging effects. These factors affect measurement stability and often require additional compensation and calibration procedures.

The reviewed studies also show that practical sensing systems usually include components beyond the FBG element itself. Depending on the application, these may include interrogation units, temperature-compensation methods, protective packaging, multiplexing architectures, and signal-processing algorithms. As system complexity increases, requirements for calibration, maintenance, and long-term operation also increase. In addition, advanced specialty-fiber platforms, including multicore and microstructured optical fibers with controlled dopant distribution or induced chirality, may further expand the design space for future sensing systems, but they also introduce additional requirements related to fabrication reproducibility, geometrical stability, attenuation control, and compatibility with standard optical-fiber technologies [168].

Another factor influencing deployment is the application itself. In sectors such as oil and gas, aerospace, and energy systems, continuous monitoring can justify the use of more complex sensing technologies. In contrast, applications that require low cost, simple installation and minimal maintenance often rely on alternative sensing approaches, even when FBG-based systems provide technical advantages.

The comparative analysis suggests that deployment maturity is determined less by the intrinsic sensing performance of FBG technology and more by application-specific implementation requirements. Across all domains, successful deployment depends on the ability to achieve reliable integration, long-term stability, and sustainable operation under realistic conditions. These factors repeatedly emerge as the principal barriers separating laboratory demonstrations from widespread practical adoption.

## 6. Conclusions and Future Research Directions

FBG sensing systems have evolved into a versatile sensing technology with applications spanning structural health monitoring, industrial monitoring, harsh environments, biomedical systems, and chemical sensing. However, the present review shows that the rapid growth of research activity has not been accompanied by a comparable increase in peer-reviewed publications documenting long-term operation under real operating conditions. Most studies remain focused on laboratory validation or pilot-scale demonstrations, whereas peer-reviewed publications describing long-term operation under real service conditions remain limited.

A key observation emerging from this review is that sensing performance is no longer the main factor restricting the wider use of FBG technology. High accuracy, multiplexing capability, and multi-parameter sensing have already been demonstrated in many application areas. The remaining challenges are largely related to deployment, including sensor integration, packaging, calibration stability, interrogation architectures, environmental durability, and long-term reliability.

The maturity of FBG sensing systems differs markedly across application domains. Structural monitoring and several harsh-environment applications have progressed furthest toward practical deployment, whereas biomedical and chemical sensing applications remain dominated by laboratory-based investigations.

Future efforts should focus on technologies and methodologies that support deployment under real operating conditions. In particular, long-term field validation, reliability assessment, scalable interrogation systems, and robust integration strategies are expected to play an important role in narrowing the gap between laboratory validation and long-term operational deployment. Economic factors, including system cost, installation, and maintenance, may also influence the practical deployment of FBG sensing systems and deserve further investigation in future studies.

The findings of this review should not be interpreted as evidence that successful practical implementations of FBG sensing systems are absent. Rather, they indicate that relatively few peer-reviewed studies report verified long-term operation under real service conditions. Increasing the availability of well-documented field studies and long-term operational reports will provide a stronger basis for assessing deployment maturity and support the transition from laboratory research to well-documented field deployment.

## Figures and Tables

**Table 1:** Representative recent review articles on FBG sensing technologies and the perspective of the present review.

Primary Perspective	Scope	Primary Objective	Ref.
Application-oriented	Battery monitoring and energy storage	Review fiber-optic sensing technologies for battery management and energy storage systems	[10]
Sensor-oriented	Displacement measurement	Review recent advances in FBG-based displacement sensing technologies	[11]
Application-oriented	High-temperature sensing	Review optical fiber sensing technologies for high-temperature monitoring	[12]
Application-oriented	Biomedical and wearable sensing	Review wearable optical fiber sensors for medical monitoring	[13]
Technology-oriented	General FBG sensing technologies	Review FBG sensor design, applications, and comparison with other sensing technologies	[14]
Deployment-oriented	Multiple application domains	Evaluate the deployment maturity of FBG sensing systems using a unified evidence-based framework	This review

**Table 2:** Representative Application-Specific Limitations in Biomedical FBG-Based Sensing Systems.

Application	Measurand	Performance	Validation Environment	Deployment Maturity	Explicit Limitation	Deployment Impact	Ref.
Wearable healthcare/HCI	Respiration, heart rate, speech	High accuracy (≤2 bpm, ~96%)	Human pilot study	3	Limited clinical applicability and lack of large-scale validation	Limited clinical deployment	[21]
Minimally invasive surgery (MIS)	3D force sensing	High resolution (mN-level sensitivity)	Laboratory + ex vivo	2	Sensor size and system complexity hinder integration into surgical tools	Limited surgical deployment	[30]
Colonoscopy systems	Interaction force	Low measurement error (~2–4%)	Simulator + ex vivo	2	Does not accurately represent real tissue–instrument interaction	Reduced clinical reliability	[31]
Endoscopic surgery	Clamping and dragging forces	High precision	Ex vivo	2	Lack of direct force quantification; reliance on indirect estimation methods	Reduced force-feedback reliability	[32]
MIS forceps	Gripping force	Good sensitivity and repeatability	Ex vivo	2	Limited temperature compensation and susceptibility to cross-sensitivity	Reduced measurement robustness	[33]

**Table 3:** Key barriers limiting the practical deployment of FBG-based chemical and biochemical sensing systems.

Application Context	Measurand/ System	Environment	Deployment Maturity	Key Limitation	Deployment Impact	Refs.
Gas sensing systems (H_2_, CH_4_, CO_2_)	Coated FBG (Pd, MOF, polymers)	Lab → Field	2–4	Functional coatings + temperature cross-sensitivity	Limits long-term stability and accuracy in real environments	[39,40,41,42,43,44]
Chemical/liquid sensing (RI-based)	RI and analyte detection systems	Lab	2	Indirect transduction	Reduces selectivity and robustness	[45,46]
Multi-parameter biochemical sensing	pH, cholesterol, biochemical markers	Lab/pilot	2–3	Calibration + cross-sensitivity	Requires complex compensation and processing	[41,43,47,48,49]
Hybrid and multi-sensor systems	Interferometer + FBG/microfluidics	Lab/pilot	2–3	System complexity	Difficult integration and maintenance	[47,48,50]
Advanced optical sensing platforms	TFBG, µFBG, spectral systems	Lab	2	Signal interrogation complexity	Requires high-cost demodulation and processing	[51,52,53]
Advanced FBG structures	Etched, tapered, phase-shifted FBG	Lab	2	Fabrication complexity	Limits scalability and reproducibility	[53,54,55]
Environmental/field monitoring systems	Gas, corrosion, infrastructure sensing	Field/semi-field	3–4	Scalability + infrastructure cost	Limits large-scale deployment	[56,57]
Cross-domain observation	Multiple applications	Lab → pilot	2–3	Limited field validation	Prevents transition to field deployment	[58,59,60,61]

**Table 4:** Systematic Synthesis of Barriers to FBG Deployment Across Structural Domains.

Application Context	Environment	Deployment Maturity	Key Limitation	Deployment Impact	Refs.
Bridge SHM	Field/operational	Level 4, with isolated Level 5 evidence	Temperature cross-sensitivity; hybrid FEM/AI dependency; lack of lifecycle validation	Limits standalone reliability and prevents fully autonomous SHM systems	[70,71,72,73,74,75,76]
Concrete Structures	Laboratory/component	Level 2–3 (lab-dominated)	Lack of field validation; embedding difficulty; durability uncertainty	Prevents structural-scale deployment and real-world validation	[45,57,58,60,61,62]
Transportation Infrastructure	Field/pilot	Level 3–4 (pilot to partial field deployment)	Installation complexity; durability issues; data dependency (AI); missing data	Limits scalability and long-term operational robustness	[67,86,87,88,89,90]
Underground/Geotechnical Systems	Field/harsh environments	Level 4 (project-specific field deployment)	System complexity; calibration drift; data management challenges	Affects long-term reliability and increases operational complexity	[95,96,97]
Advanced Optical/Sensor Systems	Laboratory/prototype	Level 1–2 (concept and lab validation)	High cost; system complexity; lack of field validation	Limits transition from sensing technology to deployable SHM systems	[100,101,102]

**Table 6:** Key limitations restricting real-world deployment in harsh-environment FBG sensing.

Application Context	Measurand/ System	Environment	Deployment Maturity	Key Limitation	Deployment Impact	Refs.
Structural health monitoring (SHM), pipeline, nuclear systems	Strain, deformation, corrosion sensing systems	Lab/simulated environments	2–3	Limited validation on scaled models or controlled setups	Prevents transition to real-system deployment due to lack of validation under operational variability	[139,140,152,153]
Oil and gas sensing and cryogenic systems	Pressure–temperature sensors, force sensing systems	Laboratory only	2	Deployment gap (no real system implementation)	Systems remain proof-of-concept; lack of field or operational adoption	[154,155]
High-temperature sensing, industrial monitoring	Temperature (750–1100 °C), high-temp strain sensing	Lab + short-term field tests	2–4	Lack of long-term stability and reliability evidence	Limits trust in continuous operation and prevents large-scale industrial adoption	[136,150,156]
Interrogators, multiplexed sensing systems	Wavelength demodulation, multi-point sensing architectures	Lab/testbed	2–3	High system complexity (hardware + signal processing)	Reduces robustness, increases cost, and complicates real-world deployment	[157,158]
Multi-parameter sensing systems	Pressure + temperature coupled sensing	Lab	2	Cross-sensitivity between measured variables	Requires complex compensation, reducing measurement reliability in real environments	[159]
Harsh-environment sensing (sewer, distributed systems, corrosive media)	Dew point, strain, temperature sensing with coatings/packaging	Lab	2	Packaging and environmental robustness limitations	Sensor performance depends on protective layers, introducing degradation and reliability concerns	[148,158]

**Table 7:** Evolution of reported temperature sensitivity in FBG-based sensors (2015–2026).

Year	Sensor Type	Sensitivity	Unit	Range	Environment	Notes	Refs.
2015–2016	Regenerated Fiber Bragg Grating (RFBG)	15.0–15.2	pm/°C	25–1000 °C	High-temperature/industrial environments	Early adoption of RFBG with enhanced thermal stability and reliable operation at elevated temperatures	[160,161]
2017–2018	Regenerated Fiber Bragg Grating (RFBG)	11–17 (temperature-dependent)	pm/°C	18–1000 °C	Aerospace/high-temperature	Temperature-dependent nonlinear sensitivity (S = 11.07 + 0.00781T)	[162]
2019–2020	FBG-based fiber laser sensor (femtosecond-inscribed)	15.9	pm/°C	300–1000 °C	High-temperature industrial/aerospace environments	Improved signal-to-noise ratio (SNR) and measurement stability using fiber laser interrogation	[163]
2021–2022	Regenerated Fiber Bragg Grating (RFBG) with enhanced packaging	15.7	pm/°C	100–1000 °C	High-temperature industrial environments	Dual-parameter sensing (temperature/strain) with high linearity and improved measurement accuracy	[164]
2023–2024	FBG-based sensors (femtosecond-inscribed/metal-coated RFBG)	15.05–15.64	pm/°C	−50–1100 °C	Harsh/industrial environments	Focus on robustness, coating techniques, and distributed sensing capabilities	[165]
2025–2026	FBG-based sensors (RFBG/femtosecond-inscribed FBG)	12.62–18.12	pm/°C	Room temperature to ~1100 °C	High-temperature/nuclear-like environments	Emphasis on linearity, stability, and performance under thermo-mechanical stress	[166,167]

## Data Availability

The data presented in this study are available from the corresponding authors upon reasonable request.
